# Molecular mechanism of interaction between fatty acid delta 6 desaturase and acyl-CoA by computational prediction

**DOI:** 10.1186/s13568-022-01410-0

**Published:** 2022-06-09

**Authors:** Jie Cui, Haiqin Chen, Xin Tang, Hao Zhang, Yong Q. Chen, Wei Chen

**Affiliations:** 1 State Key Laboratory of Food Science and Technology, School of Food Science and Technology, Jiangnan University, Wuxi, 214122 People’s Republic of China; 2 School of Food Science and Technology, Jiangnan University, Wuxi, 214122 People’s Republic of China; 3 National Engineering Research Center for Functional Food, Jiangnan University, Wuxi, 214122 People’s Republic of China; 4 Wuxi Translational Medicine Research Center and Jiangsu Translational Medicine Research Institute Wuxi Branch, Wuxi, 214122 People’s Republic of China; 5 Department of Cancer Biology, Wake Forest School of Medicine, Winston-Salem, NC 27127 USA

**Keywords:** Desaturation, Ligand-binding site, Molecular docking, Pose generation and evaluation, Modeling

## Abstract

Enzyme catalyzed desaturation of intracellular fatty acids plays an important role in various physiological and pathological processes related to lipids. Limited to the multiple transmembrane domains, it is difficult to obtain their three-dimensional structure of fatty acid desaturases. So how they interact with their substrates is unclear. Here, we predicted the complex of *Micromonas pusilla* delta 6 desaturase (*Mp*FADS6) with the substrate linoleinyl-CoA (ALA-CoA) by trRosetta software and docking poses by Dock 6 software. The potential enzyme–substrate binding sites were anchored by analysis of the complex. Then, site-directed mutagenesis and activity verification clarified that W290, W224, and F352 were critical residues of the substrate tunnel and directly bonded to ALA-CoA. H94 and H69 were indispensable for transporting electrons with heme. H452, N445, and H358 significantly influenced the recognition and attraction of *Mp*FADS6 to the substrate. These findings provide new insights and methods to determine the structure, mechanisms and directed transformation of membrane-bound desaturases.

**Graphical Abstract:**

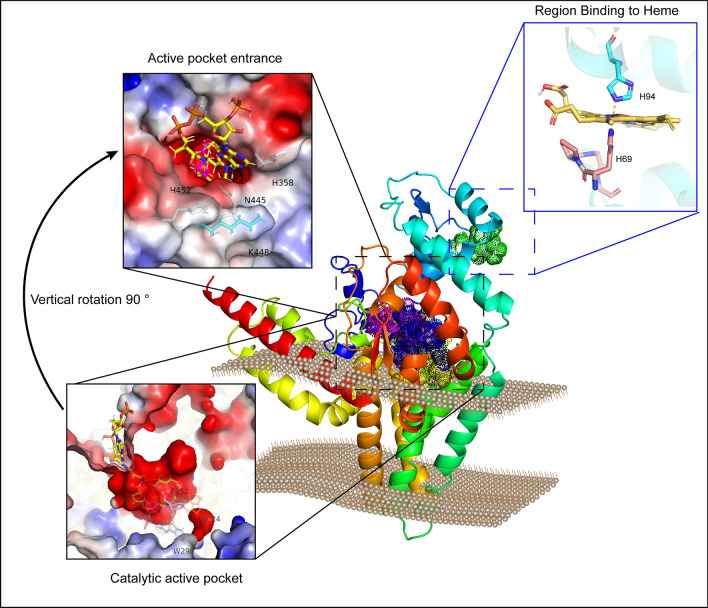

**Supplementary Information:**

The online version contains supplementary material available at 10.1186/s13568-022-01410-0.

## Keypoints

- The structure of the Δ6 fatty acid desaturase and substrate complex is modeled.
- The substrate tunnel and key residues of *Mp*FADS6 catalytic activity are determined.
- The new insights to determine the mechanism of the membrane-bound desaturases.

**Supplementary Information:**

The online version contains supplementary material available at 10.1186/s13568-022-01410-0.

## Introduction

In vivo, membrane proteins are involved in various essential signal transduction, synthesis, and metabolism events, such as the transport of ions and small molecules, and intercellular and intracellular communication. They are also important drug targets. Approximately 60% of current drugs target membrane proteins (Wang et al. 2020). As a membrane-bound protein, fatty acid desaturase is a necessary component of anabolic long-chain polyunsaturated fatty acids (PUFAs). Numerous studies have illustrated that the ratio of omega-6/omega-3 PUFAs is associated with physiological health (Tosi et al. 2014). Delta 6 fatty acid desaturase (FADS6) is the first step in the metabolic pathway of omega-6/omega-3 PUFAs. The activity and preference of FADS6 directly affect the ratio of omega-6/omega-3 PUFAs. It catalyzes the conversion of linoleic acid (LA) to gamma linolenic acid (GLA) and of linoleic acid (ALA) to stearidonic acid (SDA). Among these enzymes, *Micromonas pusilla* delta 6 desaturase (*Mp*FADS6) reportedly showed a greater preference for ALA (omega-3 PUFA) than LA (omega-6 PUFA) (Shi et al. 2015). Molecular analysis of fatty acid desaturases that have significant differences in sequence and substrate specificity in various organisms will promote the understanding of their roles in physiological regulation.

However, the membrane-bound fatty acid desaturases have multiple transmembrane domains that fail to be expressed in *Escherichia coli*. The hydrophobic surface of fatty acid desaturases hinders detergents from separating and dissolving proteins. These facts have hindered the study of the interactions of desaturases with their substrates. The complete lack of structural data has also complicated the sequence-based analysis of the desaturation mechanism. In 2015, the crystal structures of integral membrane stearoyl-CoA desaturase from humans (Wang et al. 2015) and musculus (muSCD1) (Bai et al. 2015) were reported, which advanced investigation of the fatty acid desaturation mechanism. In 2020, the structure of active muSCD1 was resolved (Shen et al. 2020). However, the crystallization of membrane-bound desaturases remains an arduous process. Molecular simulation provides a unique method to research the structure of membrane proteins, providing data that complements experimental findings.

The confidence of membrane protein structure prediction increases according to the numbers of membrane protein structures in the database used for derivation and verification (Dowhan et al. 2019; Du et al. 2020; Yang et al. 2020). Compared to soluble protein modeling, approaches for membrane proteins lack tentative structures, which has delayed the development of algorithms and their application. Modeling of membrane proteins mainly focuses on attaining models of unknown proteins without template structures. The mainstream modeling technology mainly comprises homology modeling, folding recognition technology, and ab initio modeling. Homology modeling is suitable for identifying amino acid sequences between query and template higher than 30%. The amino acid sequence identity among the subfamilies of fatty acid desaturases is below this threshold. However, it was recently reported that the root-mean-square deviation (RMSD) of the G protein-coupled receptor homology model and the actual structure was 2.9 Å, and its sequence identity was only 15% of the template (Chen et al. 2014). Folding recognition technology generates low-resolution protein models for known structures as low as 5–25%. The accuracy of these models rarely reaches an RMSD of 3–4 Å (Alford et al. 2015). Because many membrane-binding proteins lack a useful template for modeling, the ab initio method might be advantageous or restrictive (Wang and Barth 2015; Yang et al. 2020).

Based on the membrane-binding protein model, molecular simulations have been used to constrain and refine the structures (Melcr et al. 2020). In addition to structural refinement, molecular simulation is also applied to determine the ligand-binding sites of proteins, which are the amino acid residues at specific positions of proteins that interact with ligands. The identification of ligand binding sites can further the understanding of the mechanism of molecular interactions and the pathogenesis of diseases, which provides insights for drug discovery and design (Altschul et al. 1990). Wang et al. classified the ligand-binding site detection methods into four categories and discussed their scope of applications (Zhao et al. 2020). It has been reported that the programs LeDock and rDock showed better performance on ligands with fewer than 10 rotatable bonds (Wang et al. 2016). However, the direct action of fatty acid desaturase causes the substrate fatty acyl-CoA to have more than 40 flexible bonds, such as the substrate molecule ALA-CoA of FADS6. It is still important to evaluate and compare the performance of these programs for fatty acid desaturase.

In this study, the muSCD1 crystal structure, the only membrane-bound fatty acid desaturase with activity in the RCSB Protein Data Bank (PDB), was used to evaluate the performance of six modeling methods and docking programs to predicted the complex of *Mp*FADS6 with the substrate linoleinyl-CoA (ALA-CoA). The potential binding sites were anchored by analysis for the complex. The contributions of these potential binding residues to the desaturation of *Mp*FADS6 were verified by alanine substitution.

## Materials and methods

### Protein sequence and reference structure

Amino acid sequence and crystal structure data of muSCD1 were obtained from the PDB (PDB ID: **4YMK**). The amino acid sequence of *Mp*FADS6 **(**Uniport ID: **C1MMV2)** was obtained from the Uniport. Ligand molecules, heme (PubChem CID: 26,945), ferrous ion (PubChem CID: 27,284), and stearoyl-CoA (SA-CoA, PubChem CID: 94,140) were loaded from the NCBI compound structure library. Substrate linoleinyl-CoA (ALA-CoA) was constructed by Chimera (version 1.13.1) (Forli et al. 2016) based on SA-CoA. The ligand was prepared as described by Chimera. Multiple sequence alignments were performed using Clustal Omega (Madeira et al. 2019).

### Modeling methods

In this study, muSCD1 was selected as the target protein to screen the fatty acid desaturase modeling method. The SCD_SW model was generated by the Swiss-model (Waterhouse et al. 2018) (https://swissmodel.expasy.org/interactive) online comparison and manual selection of templates. The SCD_CI and SCD_CA models were automatically generated by the C-I-TASSER (Yang and Zhang 2015) (https://zhanglab.ccmb.med.umich.edu/C-I-TASSER/) and CATHER suites (Du et al. 2020) (https://yanglab.nankai.edu.cn/CATHER/), respectively. ROBETTA (http://new.robetta.org/), C-QUARK (Zheng et al. 2019) (https://zhanglab.ccmb.med.umich.edu/C-QUARK/), and trRosetta (Yang et al. 2020) (https://yanglab.nankai.edu.cn/trRosetta/) were used as ab initio modeling methods to build models SCD_RB, SCD_CQ, and SCD_trR, respectively. The *Mp*FADS6 model was constructed using trRosetta. The subsequent mutation models were based on the *Mp*FADS6 model, built and optimized for energy by Chimera.

### Model evaluation

Structural models were evaluated using QMEAN (Benkert et al. 2011) (https://swissmodel.expasy.org/qmean/) and SAVES (Laskowski et al. 1993) (https://servicesn.mbi.ucla.edu/SAVES/). In addition, to assess whether these modeling methods are suitable for fatty acid desaturases, two important indices were introduced to evaluate model accuracy (Martí-Renom et al. 2000): the degree of conservation (RMSD of conserved residues) and the correctness of alignment (TM score and global RMSD) between the target and template in Table 1. Using mTM-align (Dong et al. 2018) (https://yanglab.nankai.edu.cn/mTM-align/) to align all models of SCD1 and the crystal structure obtained by X-ray diffraction at a resolution of 2.60 Å, the TM score and structure-based phylogenetic tree were generated. The RMSD of the SA-CoA binding pocket in muSCD1 is also an important indicator calculated using PyMOL (version 2.3.4).

**Table 1:** Evaluation of the overall difference between the constructed model and the crystal structure of muSCD1

	TM-score^a^	Global RMSD^b^	RMSD of tunnel^3^	Model method
muSCD1	1.0000	0.0000	0.0000	PDB ID 4YMK
SCD_CI	0.9958	0.4470	–	C-I-TASSER
SCD_trR	0.8296	3.0400	0.7240	trRosetta
SCD_RB	0.6478	0.4980	3.8030	Robetta
SCD_CQ	0.4148	4.5180	–	C-QUARK
SCD_SW	0.3342	4.7420	–	Swiss-model
SCD_CA	0.2798	6.6120	–	CATHER

^a^TM Score is an index used to detect the structural similarity between two proteins. The TM Score is between 0 and 1, where 1 indicates that the two are completely consistent

^b^The global RMSD value measures the degree of difference between the two structures

^c^The RMSD of the residues composed of the substrate tunnel indicated the difference between model and crystal. The amino acid selected here is consistent with Fig. 4D

– means this model was not compared

### Molecular docking

Based on the classification of released hotspot prediction servers, the FTMap (Kozakov et al. 2015) (https://ftmap.bu.edu/home.php) were used to predict the potential binding sites.

The docking procedures with the specific parameters are listed as follows:

For AutoDock Vina (version 4.2.6) (Morris et al. 2009).

AutoDockTools were used to convert the ligand and receptor files into the pdbqt format and specify them as ligand and macromolecule, respectively. The docking box was configured, the configuration file was created, and AutoDock Vina was launched.

For LeDock (version 1.0) (Zhang and Zhao 2016).

The command *lepro* was used to process the receptor protein and set the docking box. The docking program was run using the command *ledock dock in*.

For DINC (http://dinc.kavrakilab.org/) (Antunes et al. 2017).

The online docking service requires an input receptor structure file, ligand structure file, and corresponding parameters.

In these three programs, the docking box was determined using the spatial range formed by histidine conserved regions in FADS6, the protein–ligand binding hotspots to predict the spatial range of services, and the cavity of the protein structure.

For rdock, dock6, and Rosetta, the docking program was run according to the respective manuals. The docking box was determined by the program.

All RMSD values were calculated using PyMOL.

### Alanine substitution

Twelve mutants of the *Mp*FADS6 were constructed using site-directed mutagenesis. The corresponding primers for each mutant were synthesized by BGI Genomics (Shenzhen, China) and are listed in Additional file 1: Table S1. The pYES2-*Mp*FADS6 plasmid was used as the template. PCR was performed in a total volume of 50 μL containing 5 μM dNTP, 5 μM 10 × buffer, 2 μM Mg^2+^, 0.5 μM of each primer, 5 ng of template plasmid DNA, and 1 U of KOD DNA polymerase. Cycling conditions involved an initial denaturation at 95 °C for 10 min, followed by 28 cycles of denaturation at 95 °C for 30 s, annealing at a temperature gradient (58–74 °C according to primers) for 30 s, extension at 68 °C for 5 min, and a final extension at 68 °C for 10 min. Amplification quality was controlled by electrophoresis. The amplicon was digested with *DpnI* at 37 °C for 1 h to exclude parental non-mutated methylated plasmid DNA and transformed into competent *E. coli* DH5α cells. Plasmid from transformants was sequenced to confirm correct mutation (BGI Genomics).

### Protein expression and whole-cell biotransformation

*S. cerevisiae* INVSc1, a strain that does not contain fatty acids C18:2 and C18:3, was transformed with the pYES2-*Mp*FADS6 wild-type and mutant vectors. Recombinant culture and induction conditions based from previous work **(**Cui et al. 2022**)**. After 8 h, the cells were treated with exogenous fatty acid substrates (0.25 mM ALA) for 12 h at 28 °C and harvested by centrifugation.

### Chromatographic analyses

The method was modified and optimized according to a previously described method **(**Shi et al. 2015**)**. The induced strains were lyophilized, and 1 mL of hydrochloric acid methanol and 20 μg pentadecanoic acid was added to 50 mg of freeze-dried cells. After methyl esterification at 70 °C for 3 h, 1 mL of saturated sodium chloride was added, shaken, and mixed. Then, 1 mL of n-hexane was added to extract fatty acid methyl esters, and the upper n-hexane layer was extracted by centrifugation at 2000 ×*g* for 5 min. Fatty acid methyl esters were then analyzed by gas chromatography-mass spectrometry (GC–MS) as previously described **(**Zhang et al. 2012**)**.

### Statistical analysis

Desaturase activity is represented by the conversion rate of ALA to SDA, CR_ALA_ = (SDA)/((ALA) + (SDA)) × 100%. All experiments were set up in three groups, and all data were statistically analyzed using SPSS Statistics 20 software (IBM, Armonk, NY, USA). The results are presented as mean ± standard deviation, with significance indicated at α < 0.05.

## Results

### Comparison and construction of membrane-bound fatty acid desaturase model

As the only active fatty acid desaturase complex crystal structure, the muSCD1_SA-CoA complex was parsed by X-ray diffraction. *Mp*FADS6 is a membrane-bound desaturase that displays < 20% identity with proteins in the PDB and only 14.9% identity with muSCD1 (Additional file 1: Fig. S1). Therefore, it is necessary to compare the models constructed using different modeling programs to obtain a highly accurate fatty acid desaturase model.

**Fig. 1:**
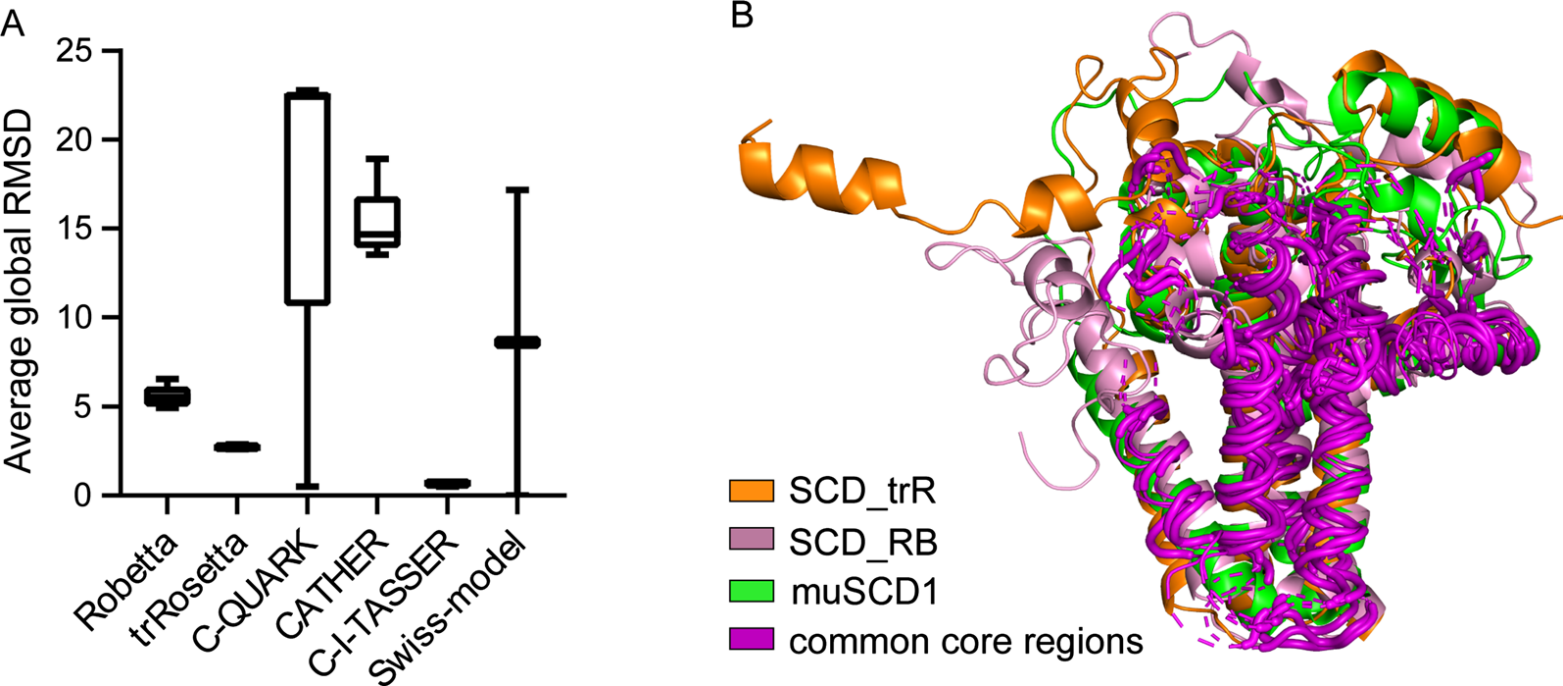
Comparison between the structure of muSCD1 and models constructed using different modeling methods. **A** The average global RMSD between the models was constructed using the six modeling methods and the data from the crystal. **B** Alignment of muSCD1 and models. The models with a global RMSD < 2 Å compared with muSCD1. The purple cartoon is a common core region with three structures

The SCD_SW homology model was constructed using the Swiss-model, which used the template manual screened in PDB. Models SCD_CI, SCD_CA, SCD_RB, SCD_CQ, and SCD_trR were auto-generated by C-I-TASSE, CATHER, Robetta, C-QUARK, and trRosetta, respectively. The global RMSD between the crystal and the five candidate models built by each program is shown in Fig. 1A. The model SCD_CI was closest to the crystal. However, this method relies on multiple templates or protein fragments to construct the final model and the 4YMK template was used to construct the SCD_CI. To fit FADS6, the following comparison excluded models that used 4YMK as a template. SCD_RB and SCD_trR were closer to muSCD1 in Fig. 1A. The TM-scores of both were > 0.5, indicative of similarity with the crystal (Table 1). Alignment of muSCD1, SCD_RB, and SCD_trR (Fig. 1B) indicated that the RMSD of the transmembrane domains of these three models was < 1 Å, indicating that they were structurally conserved regions. The conformational consistency of these domains was higher than that of the cytoplasmic region interacting with the substrate. Although the global RMSD of SCD_trR reached 3.04 Å, the TM score showed that SCD_trR was highly similar to muSCD1. RMSD is the average distance between atoms of the system, and it seems that the loose regions in the overall protein, especially the N- or C-terminus, increased the global RMSD value.

To explore whether SCD_RB and SCD_trR can reflect the actual structure, SA-CoA was imported into the model and the distance between the interaction sites was analyzed. In Additional file 1: Fig. S2, We compared the changes in the spatial position of the residues that interact with the substrate SA-CoA which have been reported (Bai et al. 2015). It shows that the RMSD of the substrate tunnel between SCD_trR and SA-CoA was < 1 Å, compared to 3.8 Å for the SCD_RB model (Additional file 1: Table S2). In contrast, the distance between the residues of the SCD_trR model and the crystal was short (Additional file 1: Table S2). Although there were still some differences with muSCD1, the total deviation of the residues binding to the SA-CoA head group in SCD_trR was in the range of 2 Å (Additional file 1: Table S2). Overall, the ab initio modeling method was more amenable for fatty acid desaturase than the other methods. Among the ab initio methods, the best model was constructed using trRosetta.

**Fig. 2:**
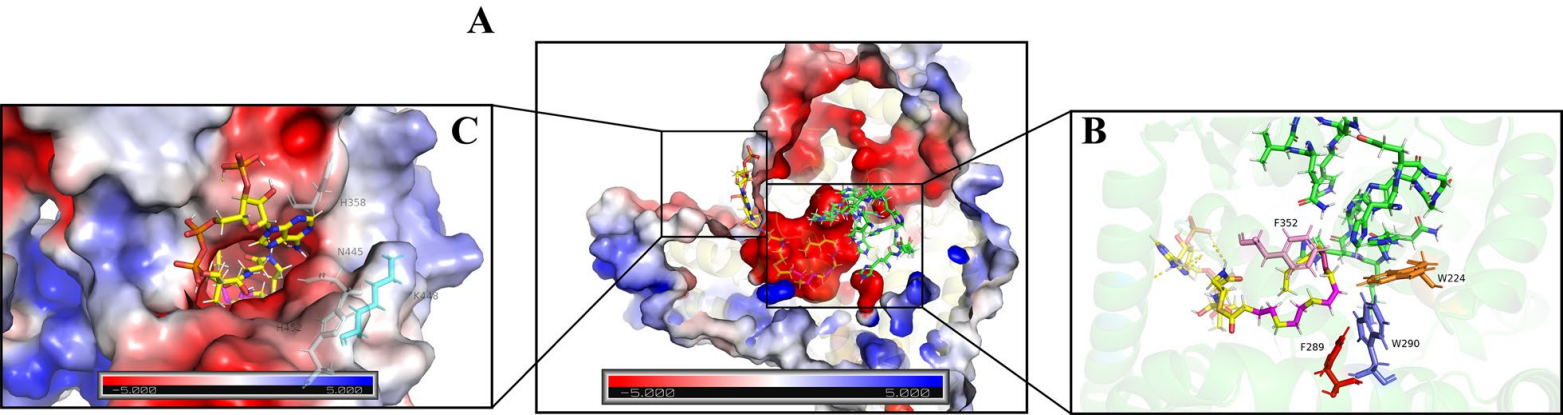
Molecular docking simulation showing the potential interaction of *Mp*FADS6 with ALA-CoA. **A** Electrostatic surface of *Mp*FADS6_ALA-CoA complex **B** Cartoon of the *Mp*FADS6_ALA-CoA complex. **C** Electrostatic surface of the head group in the *Mp*FADS6_ALA-CoA complex. The atoms are highlighted in light red in ALA-CoA, indicating the sixth and seventh carbons from the methyl end. The atoms highlighted in magenta indicate the position of the fatty acid unsaturated bond

Therefore, the *Mp*FADS6 model (Fig. 2) was constructed using trRosetta and the quality was evaluated using QMEAN and PROCKER. As shown in Additional file 1: Table S3, 99.0% and 100% residues of the *Mp*FADS6 model were in the most favored and additional allowed regions, respectively (Additional file 1: Fig. S3). QMEAN scores were accepted as − 3.87. It indicated that the *Mp*FADS6 model constructed by trRosetta were closest to the real confirmation and displayed satisfactory quality. Therefore, the *Mp*FADS6 model was able to explore substrate binding sites.

### Docking to explore potential binding sites of delta 6 fatty acid desaturase with the substrate

The *Mp*FADS6 model in the present study had satisfactory stereochemical quality and was used for the docking of the substrate. However, the ligand head group of fatty acyl-CoA with higher flexibility caused the difficulty of docking to fatty acid desaturase. After evaluating the five mainstream academic docking procedures, it was found the best pose was obtained by dock6 in which the acyl chain extended well into the hydrophobic cavity in a reasonable range and formed a stable state with the key binding residues of fatty acid desaturase (Fig. 2 and Table 2). And LeDock and rDock showed better performance on ligand heme with few rotatable bonds (Fig. 4A and Table 2).

**Table 2:** Docking scores of *Mp*FADS6 mutants with ALA-CoA

Residue position	Mutants	Heme^a^	ALA-CoA^b^	RMSD^c^
	WT	− 4.5	− 6.01	0.000
N-terminal	H69A	− 4.9		0.140
	H94A	0.0		0.140
	S97A	− 3.9		0.140
Substrate tunnel	W224A		–	0.000
	W290A		–	0.000
	F352A		− 5.44	0.000
	F289A		− 4.99	0.000
	M223A		− 5.78	0.000
Surface	K448A		− 6.44	0.000
	H358A		− 4.02	0.000
	N445A		− 4.52	0.000
	H452A		− 5.65	0.000

^a^Affinity for the mutants with the substrate Heme by Ledock (kcal/mol)

^b^Affinity for the mutants with the substrate ALA-CoA by Dock6 (kcal/mol)

^c^The global RMSD of alignment of model *Mp*FADS6 and mutant

– means the substrate is not bound to the mutant

The acyl tail of ALA-CoA was in a negatively charged cavity surrounded by three histidine conserved regions (Fig. 2B). The head group of CoA was attracted by the positive charge and anchored to the protein surface (Fig. 2C). As previously reported, heme bonded to the cytochrome *b*_5_-like domain at the N-terminus of *Mp*FADS6 (HPGG), in which a potential interaction site revealed that heme formed a Π-Π stack with residues H69 and H94 (Fig. 4A). Figure 2B shows the potential sites where ALA-CoA interacted in the histidine conserved domains to introduce a double bond at the sixth position (highlighted in light red) from the methyl terminal. Residues H452, H358, K448, and N445 may promote substrate stability. In addition, as a preferred substrate for *Mp*FADS6, ALA is formed by dehydrogenation of LA at the fifteenth carbon atom from the methyl terminal via omega-3 desaturase. This means that the LA and ALA structures only differ by one double bond (highlighted in magenta in Fig. 2B. Residues F352, W224, M227, and F289 might strengthen the desaturation of the interaction between the double bond of ALA and these residues, resulting in substrate preference.

### Effect of mutation site on substrate binding ability

**Fig. 3:**
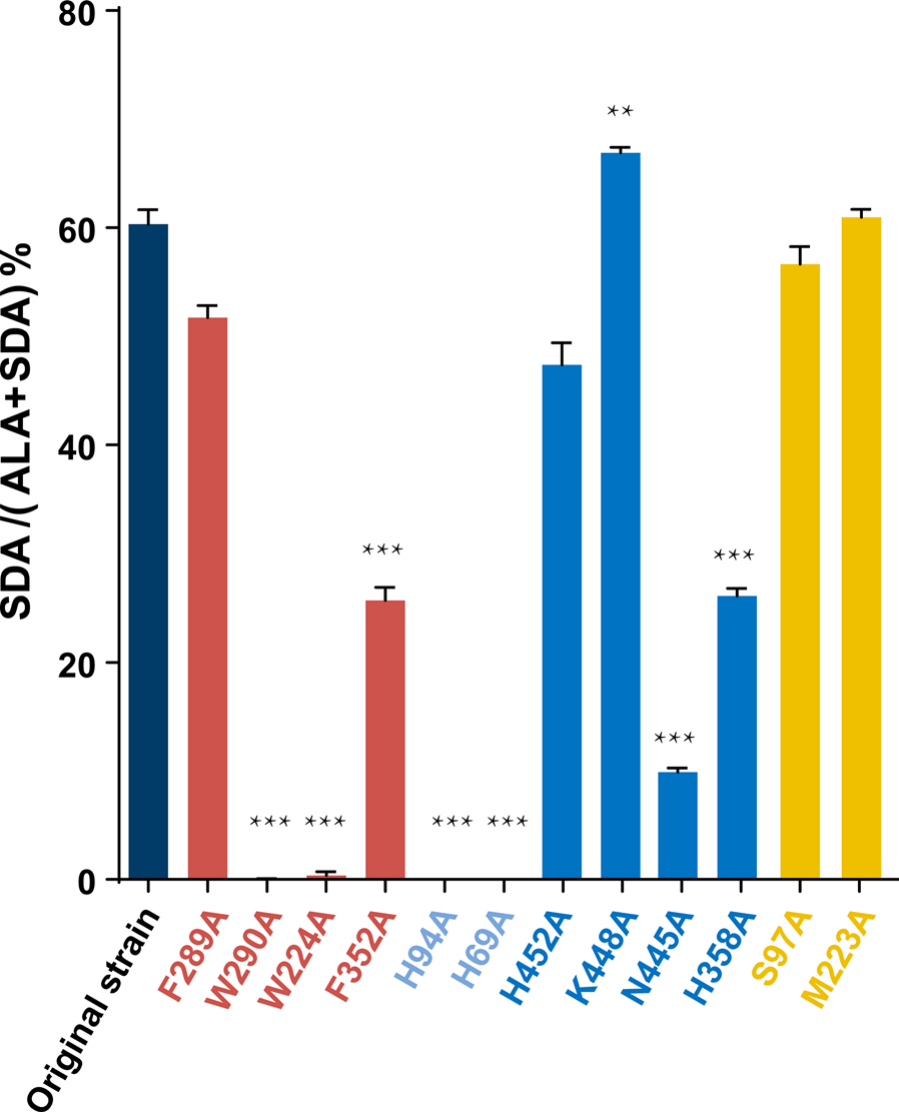
Activities of wild-type *Mp*FADS6 and mutants to ALA. The sites predicted to constitute the substrate channel of *Mp*FADS6 are indicated in red. The sites of *Mp*FADS6 predicted to bind heme are indicated in light blue. The sites of *Mp*FADS6 predicted to recognize and bind the head group of ALA-CoA is indicated in blue. Mutant obtained by accident is indicated in yellow. *p < 0.05. **p < 0.01. ***p < 0.001

To confirm the influence of the amino acid sites analyzed above on desaturation, the 10 residues were mutated to alanine, and the corresponding *S. cerevisiae* recombinants were constructed because *S. cerevisiae* does not contain ALA and SDA, which was the substrate and product of FADS6, respectively. After induction by galactose for 8 h, ALA was added as a substrate for the enzymatic reaction, and the fatty acids were extracted for methyl esterification and analysis by GC–MS. The desaturase activity was shown in Fig. 3 and Additional file 1: Table S4. The corresponding mutant models were built by Chimera and compared the structural difference with original model. And the affinities of the mutants to the substrate were analyzed by docking (Table 2). In addition to these 10 mutations by calculated analyses, two random mutations, S97A and M223A, were simultaneously analyzed for structural differences and enzyme activities.

**Fig. 4:**
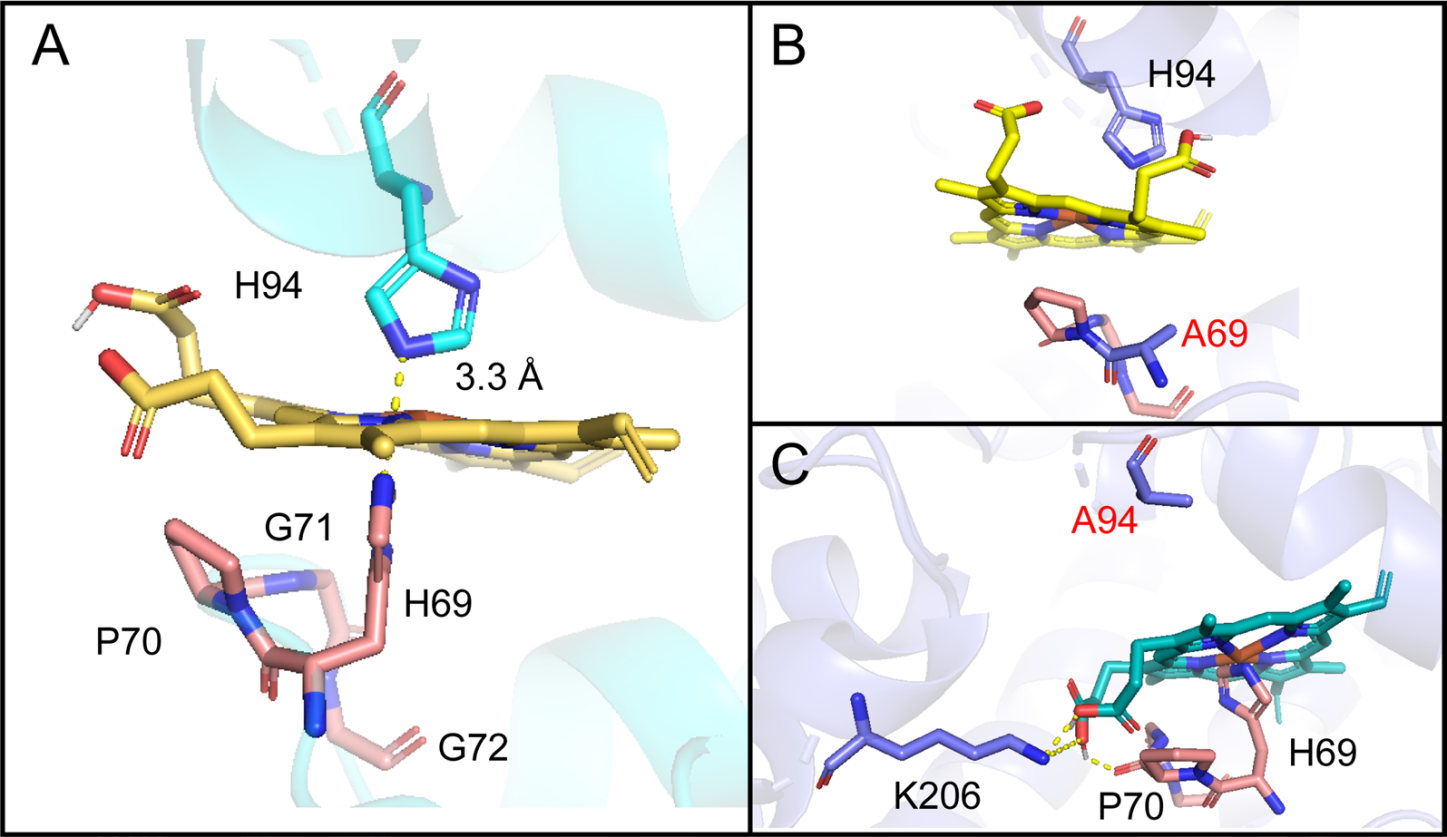
Key binding sites of *Mp*FADS6 to heme (**A**). Conformational changes in *Mp*FADS6 due to mutations H94A (**B**) and H69A (**C**). Yellow stick indicates heme with divalent iron in the center. The salmon stick indicates the Cyt *b*_5_-like domain at the N-terminus of *Mp*FADS6

As shown in Fig. 4A, H94 and H69 in the heme-binding region have the imidazole groups that attracted divalent iron at the heme center to form a stable binding. One of H94 and H69 was mutated to alanine, which suddenly lost the binding force and blocked desaturation (Fig. 3). The docking results in Table 2 show that H69A and H94A have low affinity for Heme, but the docking posts in Fig. 4B and C show that heme cannot form a stable binding with HPGG domain. However, the affinity of the random mutation S97A at the N-terminal reduced and Heme is right in the HPGG domain like Fig. 4A, so the desaturase activity retained 84.44% (Fig. 3). Therefore, H69A and H94A could not convert ALA into SDA, where electron transfer was most likely blocked because of the unstable reaction to heme.

**Fig. 5:**
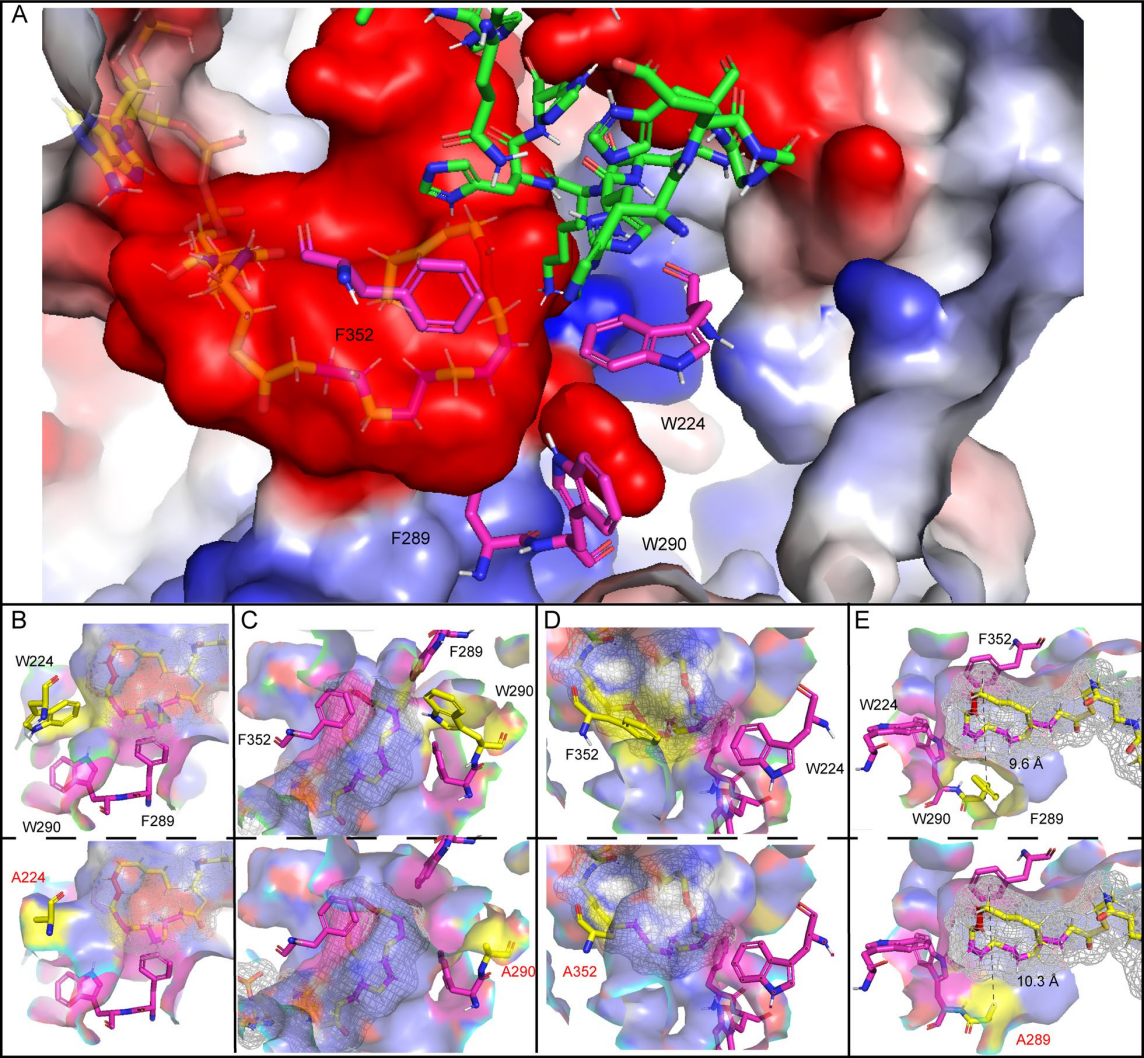
The key amino acids in the substrate cavity of *Mp*FADS6 (**A**). Conformational changes in *Mp*FADS6 due to mutations W224 (**B**), W290 (**C**), F352A (**D**), and F289A (**E**). The yellow stick indicates the mesh of substrate ALA-CoA. The red atom represents the position of the double bond induced by FADS6. The magenta atom represents the double bond at C15 of ALA

In addition, one mutant among W290A, W224A, and F352A directly bound the substrate, which led to the inactivation of *Mp*FADS6 (Fig. 3). According to the substrate mesh (Fig. 5), one of the mutants rendered the residues in the substrate tunnel incapable of contacting the substrate acyl chain, which failed to recognize the double bond of ALA-CoA and switch on desaturation. In contrast, the side chain group of F289A was distant from the substrate, which made the cavity space larger (Fig. 5E). This weakened the stability of the complex state of the protein and the substrate. The activity and affinity of *Mp*FADS6 decreased, but inactivation was not observed after the mutation of F289, in which the desaturase activity retained 85.72% (Table 2 and Fig. 3). However, the random mutation M223A has 99.95% activity of *Mp*FADS6 (Fig. 3). Molecular simulation analysis indicated that M223 was far from the cavity of the protein substrate that was not in contact with the substrate (Additional file 1: Figure S4). The conformation of W224 and W290, which constituted the substrate channel, remained unchanged during the methionine to alanine mutation. Therefore, M223A was not the key binding residue of *Mp*FADS6.

**Fig. 6:**
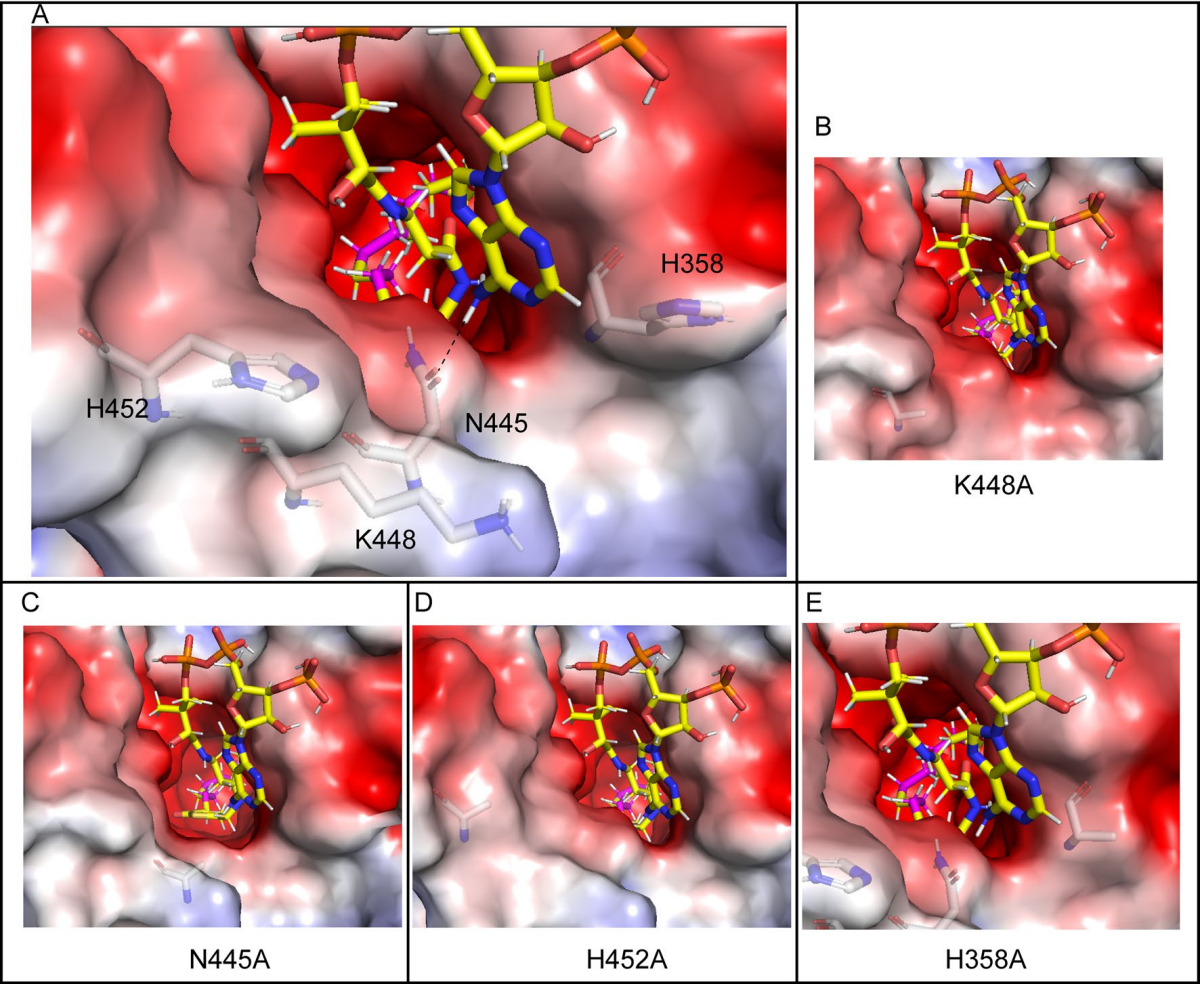
The key amino acid site for *Mp*FADS6 binding to the substrate head group (**A**). Conformational changes in K448A (**B**), N445A (**C**), N452A (**D**), and H358A (**E**). The yellow stick indicates the mesh of substrate ALA-CoA

The head group of the ALA-CoA substrate was located on the surface of the protein and had pronounced flexibility. Many residues contacted the *Mp*FADS6 surface, which made the analysis difficult. Therefore, H452, N445, K448, and H358 residues located at the entrance of the substrate cavity were selected for investigation. Alanine mutations were detected on these four amino acids (Fig. 6). The increased activity of K448A was speculated to reflect the enlarged entrance of the cavity after mutation (Fig. 6B), which increased the recognition and attraction of *Mp*FADS6 to the substrate and blocked the electrostatic attraction of lysine to the CoA group. These changes increased the probability that the substrate would enter the cavity in the correct posture. Therefore, the desaturation activity of K448A increased by 1.08 times (Fig. 3). Due to the electrostatic interaction, N445A lost its covalent connection with the adenosine group (Fig. 6C). After mutations of H452 and H358, the positively charged domains were converted to neutral (Fig. 6D and E), which weakened the protein surface and attracted the adenosine group of ALA-CoA. Thereby reducing the recognition and attraction of *Mp*FADS6 to the substrate, resulting in a significant decrease in the activity, and the retained activities are less than 50% of wild type (Fig. 3).

## Discussion

Fatty acid desaturase is composed of multiple transmembrane domains that are difficult to express and purify. The lack of structural information and the low sequence identity with other proteins make it difficult to obtain insights into the desaturation mechanism from sequence information. To date, the only active fatty acid desaturase complex crystal structure, muSCD1, was parsed by X-ray diffraction which was used in the present study to evaluate the current modeling and docking programs.

Generally, the sequence identity of proteins with different functions in the fatty acid desaturase family is < 30%, and the quality of the models constructed by homology modeling lacks confidence. As the representatives of the threading approach, C-I-TASSER and CATHER are based on multiple templates or protein fragments to construct the final model, which has been suggested to be more accurate than a single template. CASP showed that comparative modeling based on multiple templates or protein fragment recombination to construct a model has more advantages than a single template (Venclovas and Margelevicius 2005). However, in terms of membrane-bound desaturase modeling, the credibility of the models SCD_CI and SCD_CA greatly depend on the template 4YMK. In contrast, the ab initio modeling method is more suitable for fatty acid desaturases with no aligned templates to build a high-quality model. The SCD1_trR and *Mp*FADS6 models constructed by trRosetta were closest to the real confirmation and displayed satisfactory quality. The models still had defects. The local entropy accumulation, bond angle, and bond length affected the total score. However, 95% of the residues in the Ramachandran diagram remained within the allowable range, indicating their high quality. Therefore, the *Mp*FADS6 model was able to explore substrate binding sites. With the announcement of Alphafold, the Alphafold Protein Structure Database (https://www.alphafold.ebi.ac.uk/) shows mammalian FADS6 models from four species, *Danio rerio* FADS6, *Homo sapiens* FADS6, *Mus musculus* FADS6, *Rattus norvegicus* FADS6. Comparing the *Mp*FADS6 model constructed by trRosetta with these mammalian FADS6 models (Additional file 1: Table S5), the results show that the global RMSD between the *Mp*FADS6 model and the four mammalian FADS6 models is about 4.369 Å, while the average RMSD of the conserved domain is < 1.0 Å, while the sequence identities are < 25%. It suggests that the FADS6 model constructed by trRosetta is highly consistent with the model constructed by Alphafold and these methods are excellent for constructing structural models of fatty acid desaturases.

The substrate of the fatty acid desaturase is in the form of fatty acyl-CoA with about more than forty rotatable atoms and a flexible conformation to enhance the difficulty of the best pose. The manner in which the entire protein is used as the target range during docking weakens the calculation speed and increases the analysis because of redundant results. If the spatial range of interaction can be determined beforehand, these defects would be reduced. Based on geometric or energy features, FTMap was able to reveal partially enclosed cavities or shallow cavities by detecting the energy potential between the probes and the cavity, providing accurate identification of binding sites by manually analyzing the interaction between each probe and its surrounding residues. Based on reliable protein structure and reasonable and accurate docking range, the six docking procedures used in this work positioned the substrate acyl-CoA into the cavity of desaturases. The partial output poses featured excessively large-amplitude torsion, which did not interact with the residues in the substrate tunnel due to the plasticity of the long-chain substrate. Most docking poses outputted by dock6 and Rosetta entered the substrate tunnel in the correct direction and displayed stretched states. However, the flexible head group of the substrate displayed an increased RMSD with the original conformation. The heme docking molecule has a small number of rotary atoms. Autodock Suite, LeDock, and rdock provided consistent poses. These programs are convenient, fast, and accurate.

The current researches on the interaction between fatty acid desaturases and substrates and the substrate specificities of fatty acid desaturases focused on using sequence alignment combined with site-directed mutagenesis or fragment exchange technology (Li et al. 2020; Rong et al. 2019a, 2019b; Shi et al. 2015, 2018). In this work, the potential substrate interaction sites of *Mp*FADS6 were determined by docking and confirmed by site-directed mutagenesis. The changed conformation of the protein was analyzed by amino acid mutations in molecular simulations to illustrate the molecular mechanism of desaturation. By replacing the amino acid with alanine to substitute the active group on the side chain with a small-volume methyl group, which has only a small effect on the structure of the protein, the influence of the residue on the enzyme activity could be explored. It is necessary to analyze the nature of the amino acids around the mutated residue and the interaction between the mutated residue and the ligand. Some amino acid residues play a key role in the interaction with the ligand.

This study provides new insights and directions for subsequent work. However, there are still some limitations, such as whether the stability of fatty acid desaturase conformation changes after mutations or anchors the phospholipid bilayer. These aspects need to be further explored in combination with molecular dynamics simulations.

## Supplementary Information

**Additional file 1: Table S1**. The primers to construct mutants. **Table S2.** RMSD of the crucial residues between the constructed model and the crystal structure of muSCD1. **Table S3.** Evaluation of model quality. **Table S4**. Fatty acid composition of *Micromonas pusilla* delta 6 fatty acid desaturase (*Mp*FADS6) mutants. **Table S5**. Sequence and structure alignment analysis of *Mp*FADS6 models constructed by trRosetta and four mammalian FADS6 models constructed by Alphafold. **Figure S1**. Alignment of amino acid sequence between *Mp*FADS6 and muSCD1(4YMK). **Figure S2**. (A). Structure-based phylogenetic analysis of models These models are the best among those constructed by the six methods. (B). Surface map of muSCD1 substrate tunnel composed of amino acids. (C). Main amino acids in the muSCD1 substrate tunnel. (D) and (E). Binding sites of muSCD1 and head group of stearoyl-CoA. (F). N148 gap between the actual structure and the two models. **Figure S3**. Quality assessment of models. (A). Ramachandran diagram of muSCD1 crystal. (B). Ramachandran diagram of model SCD_trR. (C). Ramachandran diagram of model *Mp*FADS6 constructed by trRrosetta. **Figure S4**. Conformational changes in *Mp*FADS6 due to mutation of M223A.

## Data Availability

The authors declare that the data supporting the findings of this study are available within the article and its Additional files.

## Abbreviations

PUFA: Polyunsaturated fatty acid
FADS6: Delta 6 fatty acid desaturase
LA: Linoleic acid
GLA: Gamma-linolenic acid
ALA: Linoleic acid
SDA: Stearidonic acid
PDB: Protein Data Bank
SA-CoA: Stearoyl-CoA
ALA-CoA: Linoleinyl-CoA
GC–MS: Gas chromatography–mass spectrometry
HPGG: The cytochrome *b*5-like domain at the N-terminus of *Mp*FADS6
