# Supplementary material for: Molecular mechanism of interaction between fatty acid delta 6 desaturase and acyl-CoA by computational prediction
Source: AMB Express. 2022 Jun 9;12:69. doi: 10.1186/s13568-022-01410-0 (PMC9184693; doi:10.1186/s13568-022-01410-0)
Supplement: Supplementary file 1 — Additional file 1: Table S1. The primers to construct mutants. Table S2. RMSD of the crucial residues between the constructed model and the crystal structure of muSCD1. Table S3. Evaluation of model quality. Table S4. Fatty acid composition of Micromonas pusilla delta 6 fatty acid desaturase (MpFADS6) mutants. Table S5. Sequence and structure alignment analysis of MpFADS6 models constructed by trRosetta and four mammalian FADS6 models constructed by Alphafold. Figure S1. Alignment of amino acid sequence between MpFADS6 and muSCD1(4YMK). Figure S2. (A). Structure-based phylogenetic analysis of models These models are the best among those constructed by the six methods. (B). Surface map of muSCD1 substrate tunnel composed of amino acids. (C). Main amino acids in the muSCD1 substrate tunnel. (D) and (E). Binding sites of muSCD1 and head group of stearoyl-CoA. (F). N148 gap between the actual structure and the two models. Figure S3. Quality assessment of models. (A). Ramachandran diagram of muSCD1 crystal. (B). Ramachandran diagram of model SCD_trR. (C). Ramachandran diagram of model MpFADS6 constructed by trRrosetta. Figure S4. Conformational changes in MpFADS6 due to mutation of M223A. [file 13568_2022_1410_MOESM1_ESM.docx]

Supplementary materials

Molecular Mechanism of Interaction between Fatty Acid Delta 6 Desaturase and Acyl-CoA by Computational Prediction

Jie Cui ^1,2^,Haiqin Chen^1,2,^*,Xin Tang^1,2^,Hao Zhang ^1,2,3,4^,Yong Q. Chen^1,2,4,5^ and Wei Chen^1,2,3^

^1^ State Key Laboratory of Food Science and Technology, Jiangnan University, Wuxi 214122, People’s Republic of China; njcj26@hotmail.com (J.C.); xintang@jiangnan.edu.cn (X.T.); zhanghao@jiangnan.edu.cn (H.Z.); yqchen@jiangnan.edu.cn (Y.C.); chenwei66@jiangnan.edu.cn (W.C.)

^2^ School of Food Science and Technology, Jiangnan University, Wuxi 214122, P. R. China

^3^ National Engineering Research Center for Functional Food, Jiangnan University, Wuxi 214122, P. R. China

^4^ Wuxi Translational Medicine Research Center and Jiangsu Translational Medicine Research Institute Wuxi Branch, Wuxi 214122, P. R. China

^5^ Department of Cancer Biology, Wake Forest School of Medicine, Winston-Salem, NC 5: 27127, USA

^*^ Correspondence: [haiqinchen@jiangnan.edu.cn](mailto:haiqinchen@jiangnan.edu.cn)

**Table S1**. The primers to construct mutants

| Primer | Amino acid site | Oligonucleotide Sequence (5'-3')^1^ | Mutants |
| --- | --- | --- | --- |
| F1 | F289 | gcgTGGATCTACGTCCTGCACCCGCGGCAGGT | pYES2-*Mp*D6-F289A |
| R1 |  | AGGACGTAGATCCAcgcCGCCTGGACGAGCAGCCC |  |
| F2 | W290 | TTCgcgATCTACGTCCTGCACCCGCGGCAGGT | pYES2-*Mp*D6-W290A |
| R2 |  | AGGACGTAGATcgcGAACGCCTGGACGAGCAGC |  |
| F3 | W224 | GAGATGgcgAACCAGATGCACAATAAGCACCA | pYES2-*Mp*D6-W224A |
| R3 |  | ATCTGGTTcgcCATCTCCCCGCTCGTGGACAG |  |
| F4 | F352 | TTCGCGCACgcgTCCACGAGCCACACGCACCT | pYES2-*Mp*D6-F352A |
| R4 |  | GTGGAcgcGTGCGCGAAGAGGTACATGTACGC |  |
| F5 | M223 | GAGgcgTGGAACCAGATGCACAATAAGCACCA | pYES2-*Mp*D6-M223A |
| R5 |  | ATCTGGTTCCAcgcCTCCCCGCTCGTGGACAG |  |
| F6 | H69 | AAACTTCAAAgcgCCGGGAGGCAGCGTGATAT | pYES2-*Mp*D6-H69A |
| R6 |  | CCGGcgcTTTGAAGTTTGTGACGTCGTACTCG |  |
| F7 | H94 | TCAAGGAGTTCgcgATGCGATCGCTTAAGGCGT | pYES2-*Mp*D6-H94A |
| R7 |  | CATcgcGAACTCCTTGAACGCCTCCGTGGCGT |  |
| F8 | S97 | TCAAGGAGTTCCACATGCGAgcgTCGCTTAAGGCGTGGAAGAT | pYES2-*Mp*D6-S97A |
| R8 |  | GCATGTGGAACTCCTTGAACGCctcCGATCGCATGTGGAACTCCT |  |
| F9 | H358 | ACGAGCCACACGgcgCTCCCGGTCGTGCCCTCG | pYES2-*Mp*D6-H358A |
| R9 |  | AGcgcCGTGTGGCTCGTGGAGAAGTGCGCGAA |  |
| F10 | K448 | TCGAACTTGGATgcgGTCGGGCAGCACTACTACGTCA | pYES2-*Mp*D6-K448A |
| R10 |  | ACcgcATCCAAGTTCGAGAACGTCGCCTTCCA |  |
| F11 | N445 | GTTCTCGgcgTTGGATAAGGTCGGGCAGCACT | pYES2-*Mp*D6-N445A |
| R11 |  | TATCCAAcgcCGAGAACGTCGCCTTCCAGGCG |  |
| F12 | H452 | CAGgcgTACTACGTCAACGGCAAGGCGGAGAA | pYES2-*Mp*D6-H452A |
| R12 |  | TTGACGTAGTAcgcCTGCCCGACCTTATCCAAGTT |  |

1 Lowercase letters indicated that this amino acid site has been mutated to alanine.

**Table S2.** RMSD of the crucial residues between the constructed model and the crystal structure of muSCD1.

|  | Substrate Anchored Residues^1^ | | | | | | | Substrate tunnel-pocket^2^ | | |
| --- | --- | --- | --- | --- | --- | --- | --- | --- | --- | --- |
|  | N75 | N148 | R155 | D156 | R188 | K189 | W262 | Trp153-Trp262 | Ala112-Trp262 | Ala112-Trp153 |
| SCD_trR | - | - | -1.1 | -0.3 | -0.1 | - | 0.1 | 1.0 | -1.5 | 2.0 |
| SCD_RB | -0.3 | - | -0.1 | - | - | -0.4 | - | 9.8 | 4.7 | 1.7 |

**Table S3.** Evaluation of model quality.

|  | muSCD1 | SCD_trR | *Mp*FADS6 |
| --- | --- | --- | --- |
| Verify^1^ | 72.90% | 65.19% | 82.94% |
| Prove^2^ | 3.10% | 6.80% | 9.40% |
| Procheck^3^ | 99.60% | 99.70% | 100% |
| QMEAN^4^ | -3.67 | -2.83 | -3.87 |

1 VERIFY3D assessment of protein models with three-dimensional (3D) profiles that can be used to score the compatibility of the 3D structure model with the amino acid sequence and identify the incorrectly modeled segment in the structure.

2 PROVE is used to assess the quality of protein crystal structures and to evaluate the deviations of the atomic volumes from the standard values.

3 PROCHECK v.3.5 is used to check the stereochemical quality of protein structures. The value is the percentage of residues in most favored regions and the additional and generously allowed regions.

4 The confidence range of QMEAN should be -4 to 0, the closer to 0, the higher the confidence.

**Table S4**. Fatty acid composition of *Micromonas pusilla* delta 6 fatty acid desaturase (*Mp*FADS6) mutants

|  | C16:0 | C16:1^Δ9^ | C18:0 | C18:1^Δ9^ | C18:2^Δ9,12^ | C18:3^Δ6,9,12^ | C18:3^Δ9,12,15^ | C18:4 ^Δ6,9,12,15^ | TFA^1^ |
| --- | --- | --- | --- | --- | --- | --- | --- | --- | --- |
| Wild type | 366.24±75.74 | 672.21±76.5 | 136.84±59.14 | 339.13±75.22 | 336.57±52.06 | 33.02±5.03 | 178.01±46.6 | 262.95±58.76 | 2325±445.12 |
| F289A | 525.45±51.62 | 827.41±72.6 | 219.14±23.3 | 469.01±35.68 | 483.78±51.03 | 0±0 | 293.7±58.65 | 306.85±47.54 | 3125.37±333.63 |
| W290A | 465.22±45.33 | 792.01±69.25 | 156.12±8.68 | 392.22±43.76 | 425.68±72.83 | 0±0 | 519.1±70.64 | 4.53±4.53 | 2754.91±274.55 |
| W224A | 492.8±24.69 | 801.86±46.24 | 199.78±26.13 | 432.17±21.89 | 445.84±14.29 | 0±0 | 512.62±59.2 | 8.02±4.42 | 2893.14±156.03 |
| F352A | 489.84±37.67 | 815.25±47.1 | 158.26±14.14 | 412.78±23.62 | 445.31±35.67 | 0±0 | 380.77±39.77 | 128±7.64 | 2830.24±190.82 |
| H94A | 546.03±53.58 | 817.79±12.07 | 192.89±25.03 | 477.53±66.48 | 511.49±2.13 | 0±0 | 427.77±25.73 | 11.76±0.44 | 2985.29±163.39 |
| H69A | 460.74±57.41 | 631.01±54.6 | 158.62±18.75 | 365.22±10.83 | 388.32±26.51 | 4.53±0.78 | 399.91±94.28 | 4.82±2.05 | 2413.22±208.99 |
| H452A | 417.68±22.42 | 740.26±31.99 | 136.28±9.75 | 368.92±22.54 | 371.81±24.76 | 14.8±1.28 | 249.27±32.42 | 211.56±6.67 | 2510.62±138.93 |
| K448A | 571.31±84.62 | 837.78±64.21 | 185.63±20.86 | 458.49±38.1 | 447.56±45.83 | 64.23±17.09 | 196.57±23.96 | 363.77±57.43 | 3125.38±292.52 |
| N445A | 716.41±322.87 | 745.3±36.88 | 417.82±279.07 | 393.45±50.79 | 405.95±12.59 | 3.44±0.83 | 464.56±36.74 | 66.76±10.35 | 3213.72±728.35 |
| H358A | 405.93±10.25 | 770.56±39.99 | 135.23±7.02 | 375.91±14.95 | 395.44±22.95 | 0.85±0.14 | 307.77±26.75 | 109.38±7.71 | 2501.11±84.6 |
| S97A | 537.07±230.74 | 545.99±159.37 | 286.63±83.25 | 320.62±102.54 | 322.16±107.54 | 17.42±7.95 | 210.09±66.75 | 232.2±86.12 | 2472.23±754.65 |
| M223A | 689.12±42.76 | 1042.5±159.47 | 247.97±2.45 | 563.17±45.17 | 566.18±92.29 | 51.9±10.65 | 258.8±65.26 | 394±111.53 | 3813.68±490.88 |

1 TFA, total fatty acids.

**Table S5**. Sequence and structure alignment analysis of *Mp*FADS6 models constructed by trRosetta and four mammalian FADS6 models constructed by Alphafold

|  | Delta 6 fatty acid desaturase | | | | | Average |
| --- | --- | --- | --- | --- | --- | --- |
| Species | *Micromonas pusilla* | *Danio rerio* | *Homo sapiens* | *Mus musculus* | *Rattus norvegicus* |  |
| UniProt | C1MMV2 | Q9DEX7 | O95864 | Q9Z0R9 | Q9Z122 | ‘- |
| Sequence identity (%) | 100.00 | 24.70 | 22.76 | 23.24 | 23.00 | 23.425 |
| Global alignment (Å) | 0.000 | 3.353 | 4.731 | 4.634 | 4.758 | 4.369 |
| HPGG region (Å) | 0.000 | 0.894 | 0.896 | 0.871 | 0.873 | 0.884 |
| His I region (Å) | 0.000 | 0.376 | 0.369 | 0.373 | 0.365 | 0.371 |
| His II region (Å) | 0.000 | 0.503 | 1.140 | 0.461 | 0.463 | 0.642 |
| His III region (Å) | 0.000 | 0.446 | 0.320 | 0.446 | 0.322 | 0.384 |


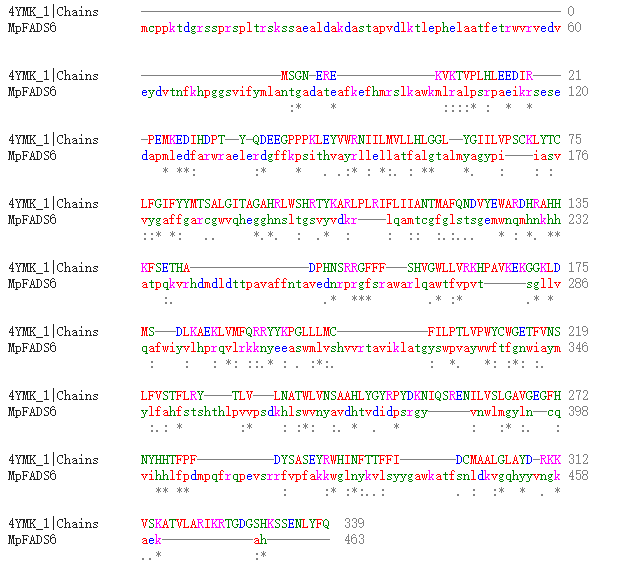


**Figure S1** Alignment of amino acid sequence between *Mp*FADS6 and muSCD1(4YMK).


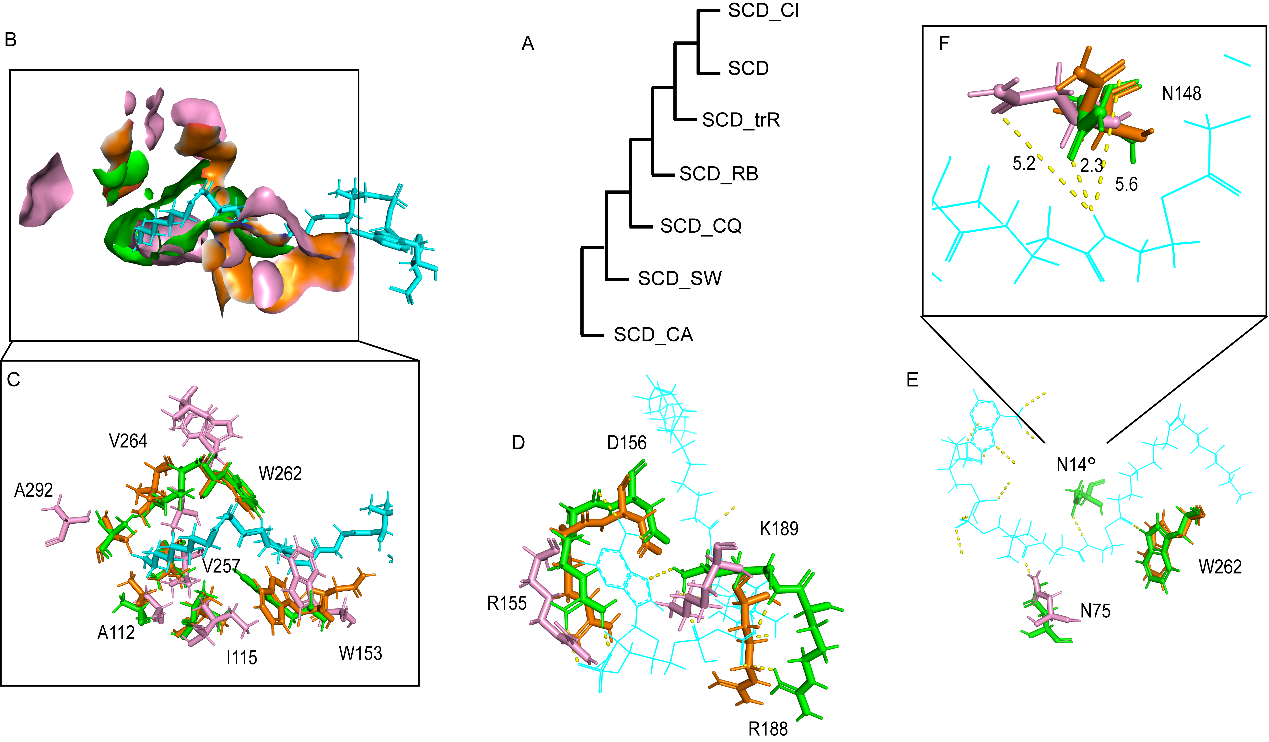


**Figure S2**. (A). Structure-based phylogenetic analysis of models These models are the best among those constructed by the six methods. (B). Surface map of muSCD1 substrate tunnel composed of amino acids. (C). Main amino acids in the muSCD1 substrate tunnel. (D) and (E). Binding sites of muSCD1 and head group of stearoyl-CoA. (F). N148 gap between the actual structure and the two models.


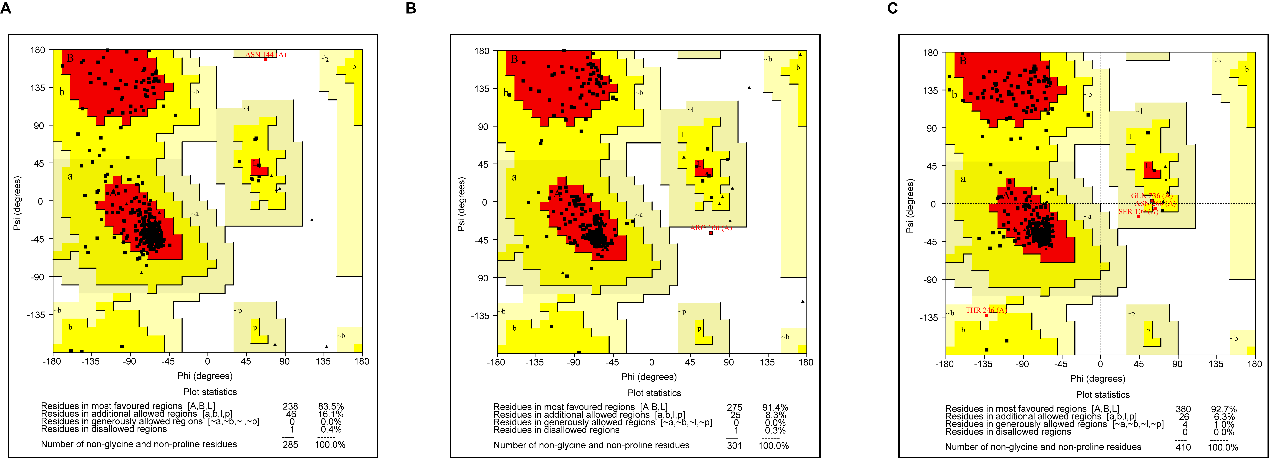


**Figure S3** Quality assessment of models. (A). Ramachandran diagram of muSCD1 crystal. (B). Ramachandran diagram of model SCD_trR. (C). Ramachandran diagram of model *Mp*FADS6 constructed by trRrosetta.


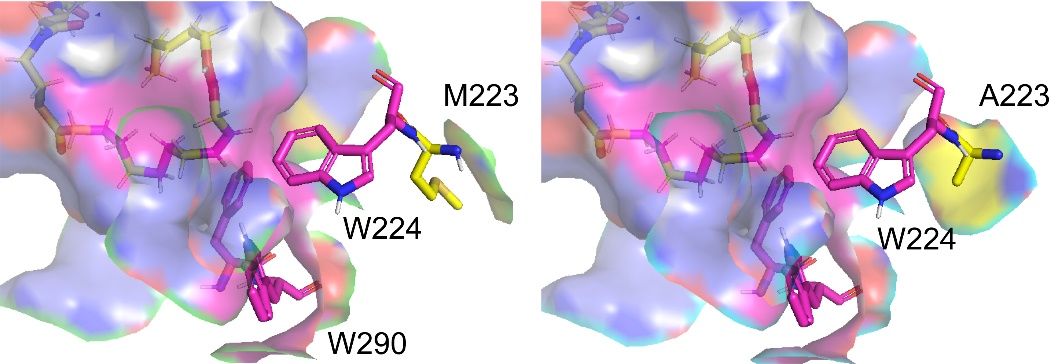


**Figure S4**. Conformational changes in *Mp*FADS6 due to mutation of M223A.
